# Myotonometric, Static Plantar Pressure, and Stabilometric Assessment in Children and Adolescents with Idiopathic Scoliosis: A Study Protocol

**DOI:** 10.3390/life16010101

**Published:** 2026-01-11

**Authors:** Oana-Cristina Rădulescu, Alina-Daniela Totorean, Oana Suciu, Andreea Niță, Liliana Catan, Alessandro Iatarola, Iuliu Șerban, Elena-Constanta Amaricai

**Affiliations:** 1 Doctoral School, “Victor Babeș” University of Medicine and Pharmacy, 300041 Timișoara, Romaniaiatarola.alessandro@yahoo.com (A.I.); 2 Department of Rehabilitation, Physical Medicine and Rheumatology, Faculty of Medicine, “Victor Babeș” University of Medicine and Pharmacy, 300041 Timișoara, Romaniacatan.liliana@umft.ro (L.C.);; 3 Research Center for Assessment of Human Motion, Functionality and Disability, “Victor Babeș” University of Medicine and Pharmacy, 300041 Timișoara, Romania; 4 Fiziokineticmed, 300158 Timișoara, Romania

**Keywords:** scoliosis, adolescent idiopathic scoliosis (AIS), myotonometry, static plantar pressure, stabilometry, exercise

## Abstract

Adolescent idiopathic scoliosis (AIS) is a 3D structural deformity of the spine that can cause decreased spinal movement, paraspinal muscle weakness, or chronic pain. Our study aims to evaluate biomechanical and viscoelastic properties of the paravertebral muscles in adolescents with idiopathic S-type scoliosis, static plantar pressure, and stabilometry at the beginning of a physical exercise program and after 3 months. The myotonometry performed by using MyotonPro will determine five parameters (frequency, stiffness, logarithmic decrement, stress relaxation time, and ratio of relaxation time to deformation time). Measurements will be taken at the level of the left/right middle trapezius, left/right lower trapezius, left/right latissimus dorsi, and left/right lumbar erector muscles. Static pressure assessment and stabilometry (length described by the center of pressure, confidence area, and speed described by the center of pressure) will be determined by a PoData device in different testing positions (eyes open, eyes closed, head rotated to the right/left, head tilted to the right/left, and head in hyperextension). We expect to record a difference between the muscles on the concave side and the convex side in terms of myotonometric parameters, as well as differences between the initial and 3-month assessment. We predict an improvement in stabilometric parameters after the 3-month physical exercise program.

## 1. Introduction

Adolescent idiopathic scoliosis (AIS) is a 3D structural deformity of the spine observed in children from 10 years of age until skeletal maturity [1]. The Scoliosis Research Society (SRS) confirms it with a Cobb angle of 10° or more accompanied by vertebral rotation [2]. The incidence of AIS ranges between 2% and 4% of adolescents in the early stages of puberty and predominantly affects females [3].

Although the exact cause and underlying mechanisms of adolescent idiopathic scoliosis (AIS) remain unclear, current research suggests it is a multifactorial disorder with significant contributions from both genetic predisposition and neuromuscular alterations. Genome-wide and genetic studies have identified various susceptibility loci and implicated multiple genetic variants in AIS risk, consistent with a polygenic model of inheritance [4]. Additionally, evidence of altered neuromuscular control—including atypical muscle activation patterns in paraspinal and scapular muscles and disrupted neurotransmission pathways—supports the hypothesis that neuromuscular dysfunction may play a role in the initiation or progression of spinal curvature [5,6]. The prevalence of scoliosis varies depending on its type and severity. It is very important to note that scoliosis can be mild and not require treatment. In moderate and severe types, it can lead to pain or breathing problems, and in severe cases it may require surgery.

Scoliosis can cause decreased spinal movement, paraspinal muscle weakness, chronic pain, psychological distress, reduced lung function, and respiratory dysfunction [7]. Idiopathic scoliosis can only be diagnosed through exclusion. It can only be claimed when historical, clinical, or radiological examinations do not reveal clear evidence of etiology. The main type of non-idiopathic scoliosis is congenital. This type is caused by defective segmentation or malformation of the neuromuscular system and vertebrae deformation due to muscle imbalance. Given that close relatives of people diagnosed with scoliosis are more prone to this condition and that the concordance in monozygotic twins goes up to 70%, it is known that at least part of the cause is genetic [8].

If untreated, progressive childhood scoliosis can lead to severe restrictive lung disease, which is a potentially life-threatening complication [6]. Sedentarism and inadequate movement can lead to postural changes in the spine, such as scoliosis [9]. Thus, prognosis depends on the severity, as well as the age and the stage of bone growth at diagnosis. In about 80% of cases, infantile scoliosis resolves spontaneously, without requiring any treatment. However, in the remaining 20% of cases, scoliosis progresses and complex, long-term treatment is necessary [8].

There are three categories of scoliosis: infantile idiopathic (0–3 years), juvenile idiopathic (4–10 years), and adolescent idiopathic (11–18 years).The last one is the most common type of scoliosis. Although it is often discovered during puberty, in many cases the curvatures are mild and do not require major treatment. If the curvatures are more severe, orthopedic treatment (braces) or even surgery may be necessary. While performing different sports, scoliosis can increase the risk of injury to the knees or ankles due to biomechanical disturbances that lead to abnormal and uncoordinated joint movements [10,11].

Currently, plantar pressure is being intensively studied as it provides information about foot posture, function, and the ability to control the whole body posture [12]. Plantar pressure results from the distribution of forces exerted by the sole of the foot on the ground while walking and standing [13]. Therefore, numerous researchers have applied plantar pressure analysis to different types of scoliosis and have observed correlations between various stabilometry parameters and different forms of scoliosis. The study of Ai et al. showed that for moderate AIS patients, static and dynamic plantar pressure parameters were primarily influenced by curve location (thoracic/lumbar). Their study adds evidence to the fact that there is a relationship between scoliosis-related pelvic tilt and foot posture. When analyzing different parts of the plantar pressure, the authors observed that the midfoot was more pronated if the scoliosis occurred at the lumber region, while it was more supinated if the scoliosis occurred at the thoracic region, especially if there was only a single curvature [13]. St-Georges et al. showed that compared to other scoliosis curve types, pressure on the right foot was associated with the degree of right thoracic Cobb angles [14]. Ai et al. stated that scoliosis patients have different compensatory mechanisms to maintain a balanced trunk [13]. More factors, such as sagittal vertical axis, should be considered in the future studies [13].

Scoliosis also influences muscle tone, elasticity, stiffness, and relaxation time. It is known that the properties of the paravertebral muscles are influenced by the convex and concave parts of scoliosis [15].

The current study protocol aims to evaluate the biomechanical and viscoelastic properties of the paravertebral muscles in adolescents with idiopathic S-type scoliosis, as well as plantar pressure and stabilometry at the initial and three-month treatment time points.

## 2. Materials and Methods

Our study will include patients with mild to moderate idiopathic S-type scoliosis (dextroconvex thoracic and sinistroconvex lumbar).

Before the study, patients should be provided with verbal and written information about the study protocol.

Patients will be selected from the Pediatric Surgery Department, Pediatric Rehabilitation Unit, at the Louis Turcanu Children’s Hospital in Timișoara, Romania. Participation in the study is voluntary. Written and oral informed consent will be obtained from all participants/their legal guardians.

The study will be conducted in accordance with the Declaration of Helsinki and was approved by the Institutional Ethics Committee of Louis Turcanu Children’s Emergency Hospital, registered with the number 173 and approved on the 13 January 2025, and the Ethics Committee of the Victor Babes University of Medicine and Pharmacy in Timișoara, registered with the number 52 and approved on the 4 November 2024.

### 2.1. Sample Size Calculation

Sample size calculations were based on the results of previous studies, employing G*Power 3.1.9.7 (Kiel University, Kiel, Germany) for matched pairs (a single group assessed before and after performing a physical exercise program). The effect size was considered to be 0.5, with type I error set at α = 0.05 and a power of 0.8. A total sample size of at least 35 patients is required. The total sample size of our study (at least 35 patients) is in accordance with that calculated by Zielinski for patients who followed kinesiotherapy with the same statistical power and effect size (36 patients) [16,17].

#### 2.1.1. Inclusion Criteria

The patients included in the studies will be children and adolescents aged between 10 and 18 years, diagnosed with mild to moderate idiopathic S-type scoliosis (dextroconvex thoracic and sinistroconvex lumbar). They must present clinical and radiological evidence of the above-mentioned spinal deviation, agree to participate voluntarily, and follow the home exercise program.

#### 2.1.2. Exclusion Criteria

Patients who have had recent (no more than 3 months ago) orthopedic surgery on the lower limbs; patients with single thoracic or lumbar scoliosis; patients with double sinistroconvex thoracic and dextroconvex lumbar scoliosis; those with static balance and gait disorders; those with a neurological pathology that impairs balance, standing posture, or gait; athletes; and those with a body mass index > 25 kg/m^2^ will be excluded. Moreover, patients will be excluded if they demonstrate deficiencies in compliance, such as the lack of ability to understand examination requirements, or if they have mental disabilities. The participants who have followed any treatment for their scoliosis (scoliosis-specific exercises with or without bracing) will also be excluded from the study.

#### 2.1.3. Discontinuation Criteria

Any patients who do not comply with the physical exercise program will be excluded from the study, as well as patients who could suffer any injury of the spine or lower limbs during the study that may affect gait or muscle strength.

#### 2.1.4. Patient Data Management Plan

The study will collect clinical and demographic data from patients with AIS. Data elements will include age, sex, weight, height, measurement parameters for myotonometry and stabilometry, physical exercise treatment regimen details, and outcome measures relevant to the study objectives. No direct identifiers (e.g., names, initials, addresses, and national identification numbers) will be included in the analytical dataset. All data will be anonymized prior to statistical analysis and dissemination of the study results. Anonymization will be performed by a licensed physician who is part of the clinical care team, before any data are transferred to the research/statistical analysis environment.

The anonymization process will include the following:

- Removal of all direct identifiers (e.g., name, medical record number, and date of birth).
- Replacement of patient identifiers with randomly generated study codes.
- Aggregation or generalization of potentially identifying variables (e.g., age reported in years rather than exact dates).
- Review of the final dataset to ensure that the re-identification risk is minimized.

The key linking study codes to patient identities will be stored securely within the clinical institution and will not be accessible to the research analysts or shared externally.

Prior to anonymization, identifiable data will be stored on secure, access-controlled institutional servers or physical patient data files (i.e., hard copy on paper) in compliance with applicable data protection regulations. After anonymization, the analytical dataset will be stored on password-protected systems accessible only to authorized members of the research team. Regular data backups will be performed in accordance with institutional policies. Upon completion of the study and publication of the results, the fully anonymized dataset will be deposited as Supplementary Material of the publication, stored by the publisher in an open-access format with long-term preservation. No restrictions will be placed on data reuse, provided users cite the original publication. No identifiable or re-identifiable patient data will be shared.

The anonymized dataset will remain publicly available indefinitely through the selected repository, ensuring long-term access and reproducibility of the study findings.

The missing-data management plan will address the following areas: anticipation and prevention, identification of mechanisms (missing at random), handling techniques and justification (imputation), documentation, and transparency. 

### 2.2. Exercise Program

The exercise program will be followed at home for 3 months, with a frequency of 5 days per week and a duration of 45 min per session. The objectives of the exercise program are the following:

- Strengthening of the muscles on the convex side of scoliosis;
- Improvement of spinal flexibility;
- Correction of curvature by decreasing the Cobb angle;
- Stretching of the muscles on the concave side of scoliosis.

The purpose of this exercise program is to correct the scoliosis or to stop its progression.

**Figure 1:**
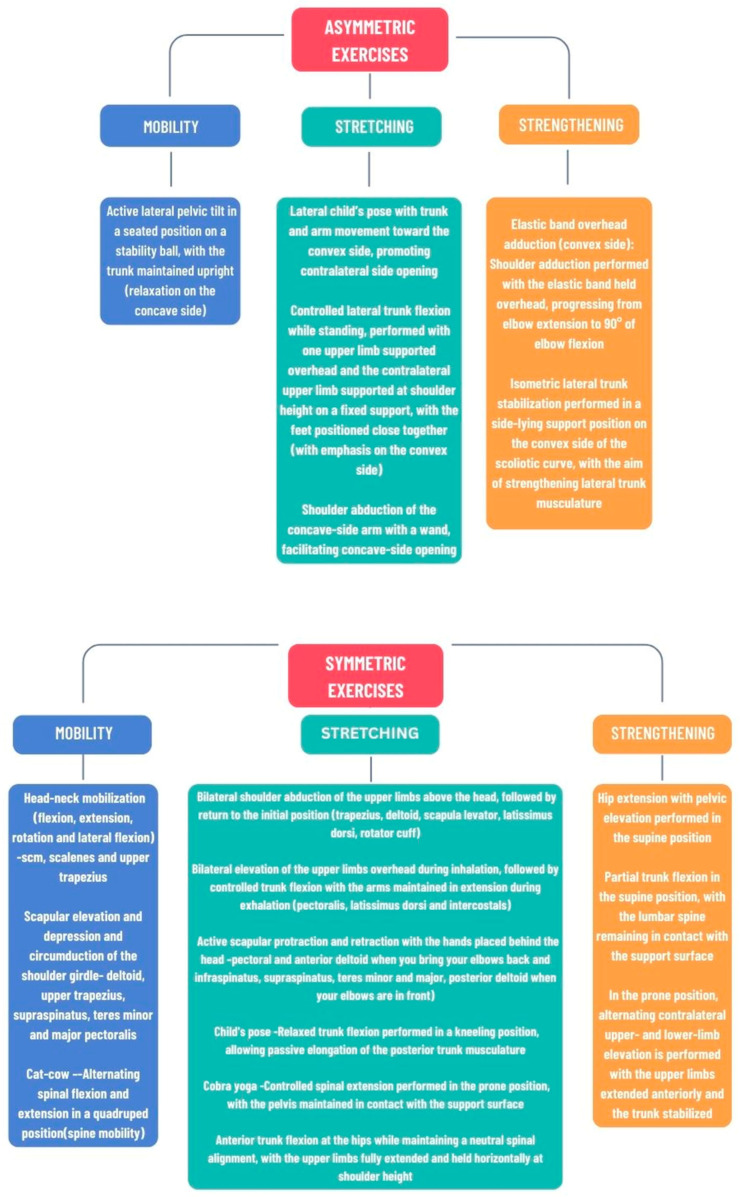
Home-based exercise program including asymmetric and symmetric exercises. Abbreviations: scm = sternocleidomastoid muscle.

For home-based sessions, participants will be instructed by a therapist on the exercises (Figure 1) and they will be asked to perform them under professional supervision until they are correctly executed. Moreover, participants and their guardians will receive written instructions and pictographic representations of the physical exercises to be performed, which are as follows:

- Weeks 1–4: Low-intensity exercises focused on mobilization and stretching, joint range of motion, and neuromuscular activation.
  - Asymmetric exercises: active lateral pelvic tilt in a seated position on a stability ball, with the trunk maintained upright (relaxation on the concave side); lateral child’s pose with trunk and arm movement toward the convex side, promoting contralateral side opening; shoulder abduction of the concave-side arm with a wand, facilitating concave-side opening.
  - Symmetric exercises: head-neck mobilization–sternocleidomastoid, scalene, and upper trapezius muscles; scapular elevation and depression, and circumduction of the shoulder girdle—deltoid, upper trapezius, supraspinatus, teres minor and major, and pectoralis; cat-cow—alternating spinal flexion and extension in a quadruped position (spine mobility); bilateral shoulder abduction of the upper limbs above the head, followed by return to the initial position (trapezius, deltoid, scapula levator, latissimus dorsi, and rotator cuff); bilateral elevation of the upper limbs overhead during inhalation, followed by controlled trunk flexion with the arms maintained in extension during exhalation (pectoralis, latissimus dorsi, and intercostals); active scapular protraction and retraction with the hands placed behind the head—pectoral and anterior deltoid when you bring your elbows back, infraspinatus, supraspinatus, teres minor and major, and posterior deltoid when your elbows are in front); child’s pose—relaxed trunk flexion performed in a kneeling position, allowing passive elongation of the posterior trunk musculature; cobra yoga—controlled spinal extension performed in the prone position, with the pelvis maintained in contact with the support surface; anterior trunk flexion at the hips while maintaining a neutral spinal alignment, with the upper limbs fully extended and held horizontally at shoulder height.
- Weeks 5–8: High-intensity muscle-strengthening exercises focused on specific muscle groups, integrated with balance and coordination training. Continue the exercise program from weeks 1–4, incorporating one additional set and including the following strengthening exercises:
  - Asymmetric exercises: elastic band overhead adduction (convex side): shoulder adduction performed with the elastic band held overhead, progressing from elbow extension to 90° of elbow flexion; isometric lateral trunk stabilization performed in a side-lying support position on the convex side of the scoliotic curve, with the aim of strengthening lateral trunk musculature.
  - Symmetric exercises: hip extension with pelvic elevation performed in the supine position; partial trunk flexion in the supine position, with the lumbar spine remaining in contact with the support surface; in the prone position, alternating contralateral upper- and lower-limb elevation is performed with the upper limbs extended anteriorly and the trunk stabilized.
- Weeks 9–12: Functional exercises performed at higher intensity levels, maintaining exercises from weeks 1–8, implementing a progressive increase to two sets of strengthening exercises. Exercise program—administered 5 times per week; each exercise repeated 10 times, with a 2 s hold at the terminal position, except for the final asymmetric strengthening plank pose, which should be held for 10 s and repeated 3 times.

### 2.3. Measurements

Patient measurements will be performed using the MyotonPro Digital Palpation Device (Myoton AS, Tallinn, Estonia) and Podata 2.0 (Chinesport, Italy) to assess muscle tone and static plantar pressure before and after the physical exercise program.

#### 2.3.1. Myotonometry

Myotonometry is a non-invasive method used to measure the mechanical properties of muscles. MyotonPro is a portable device that applies a controlled force to the muscle and records its response. The device measures (simultaneously calculates) the following five parameters:

- Tension state: natural oscillation frequency [Hz] that characterizes tone or tension state;
- Biomechanical properties: dynamic rigidity [N/m]; logarithmic decrement, which characterizes elasticity or dissipation of natural oscillation; Viscoelastic properties: mechanical stress relaxation time [ms] characterizes tissue regeneration after displacement (the higher the tension or stiffness of the tissue, the faster the tissue regains its shape, which means a lower value); ratio between relaxation time and deformation time, which characterizes creep (Deborah number) [17].

The MyotonPro device applies five mechanical pulses delivered to muscles’ center at intervals of one second (0.18 N) and compresses the subcutaneous tissue for an additional 15 ms (0.40 N). These pulses transmit data back to the accelerometer probe of the MyotonPro machinery, calculating the viscoelastic and biomechanical properties [18].

Myotonometric parameters will be measured at the concave and convex sides of the most curved paraspinal muscles [19]. For the thoracic scoliosis, we chose the trapezius and latissimus dorsi muscles based on the previous research of Gökalp et al., which included AIS patients with a thoracic curve that was convex on the right side [20].

**Figure 2:**
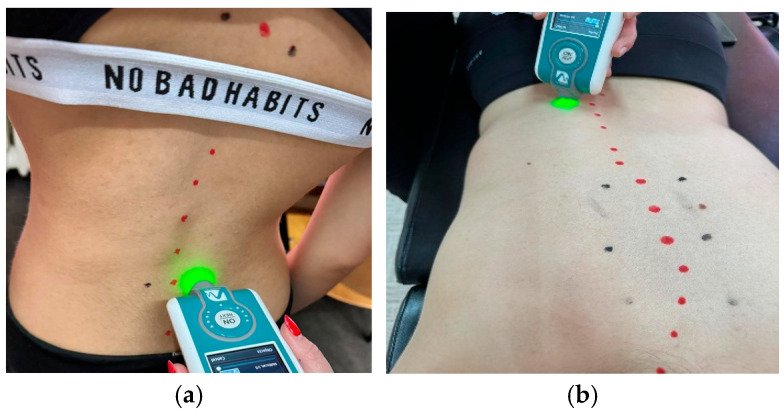
Myotonometric assessment of the spine in the standing position (**a**) and prone position (**b**) using the MyotonPro device.

Patients will be asked to lie down with their paravertebral muscles completely relaxed, arms at their sides, and head held in a neutral position with the aid of a support. The contours of the spinous processes will be traced with a marker pen. Three reference points will be noted, respectively, at the top of the curve, at the top and bottom of the main curves (thoracic and lumbar), and 2 cm lateral to the spinous process. Measurements are taken in two positions, standing and prone, at the level of the left/right middle trapezius, left/right lower trapezius, left/right latissimus dorsi, and left/right lumbar erector muscles (Figure 2).

The myotonometric parameters of the paravertebral muscles on the convex side compared to the concave side and the paravertebral muscles (convexity and concavity) in the three assessments will be analyzed.

#### 2.3.2. Static Plantar Pressure Assessment

**Figure 3:**
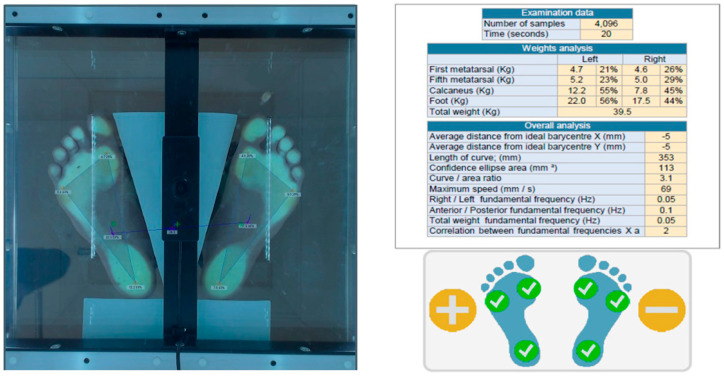
Automatically defined distal 60% of footprint length as front zone and proximal 40% as back zone assessment of static plantar pressure.

The plantar pressure and stabilometry will be assessed by employing PoDATA 2.0 (Chinesport, Italy).The patient will be positioned upright. The following testing conditions will be assessed: with eyes open, with eyes closed, head rotated to the right/left, head tilted to the right/left, and head in hyperextension. It is a practical, non-invasive method that is easy to apply. The device records the distribution of body weight at three points (for each foot): metatarsal head 1, metatarsal head 5, and heel (Figure 3). Plantar pressure analysis allows the evaluation of how plantar pressures and forces are distributed in the static standing posture.

Stabilometry determines the center of pressure in relation to the ideal for the subject. The following data are recorded: the length described by the center of pressure, the confidence area, and the speed described by the center of pressure.

### 2.4. Statistical Analysis

Descriptive statistics (mean and standard deviation) shall be computed for all recorded variables. The D’Agostino–Pearson normality test shall be utilized to verify the Gaussian distribution of values before statistical analysis. A one-way ANOVA with a Bonferroni Post Hoc test shall be applied for intragroup data comparisons (myotonometer, plantar pressure, and stabilometry parameters at baseline and after the 3-month physical exercise program). The myotonometer parameters of the convex and concave side shall be compared with Student’s unpaired t-test or the Chi-squared test. A *p*-value < 0.05 shall be considered statistically significant.

## 3. Expected Outcomes

As expected outcomes we envisage a difference between the muscles on the concave side and the convex side in terms of myotonometric parameters (resting muscle tone, elasticity, muscle stiffness, and muscle relaxation time). Differences between the initial assessment and the assessment at 3 months (such as increased resting muscle tone, increased elasticity, and decreased stiffness at the 3-month assessment) are anticipated. Regarding plantar pressure, we aim to quantify the difference between plantar pressure loading on the feet in different testing conditions (for example, with eyes open vs. with eyes closed). We will also investigate possible correlations between plantar pressure values on the right and left foot, respectively, and Cobb angle of both the thoracic curve and the lumbar curve. We anticipate an improvement in stabilometric parameters (length described by the center of pressure, area of the center of pressure ellipse, and speed of the center of pressure) after 3 months of physical exercise. The data provided by the myotonometric assessment (viscoelastic properties of the muscles and muscle stiffness) on the concave and convex sides can be used by the medical rehabilitation team for individualizing physical exercise programs.

## 4. Discussion

The present study will compare the biomechanical and viscoelastic properties of the paravertebral muscles on the convex side with those on the concave side in a particular group of patients, namely those diagnosed with AIS. The comparisons will target both thoracic and lumbar curvatures, as the included patients will have a double S-type scoliosis. We decided to include only patients with thoracic dextroconvex curvature and lumbar sinistroconvex curvature so that the data can be more easily analyzed.

Besides the comparison of the muscle properties between the convex and concave side of scoliosis, we will analyze the data of the same groups of muscles at the beginning of the physical exercise program and after 3 months of performing the exercises.

The very recent study of Ferran de la Cierva revealed good to excellent inter-rater reliability for the trapezius muscle [21]. Kurashina et al. also showed a good intra-rater and inter-rater reliability of MyotonPro for latissimus dorsi (ICC = 0.94–0.99) [22]. Another study by Valenti et al. demonstrated high intra- and inter-reliability of lumbar erector muscle stiffness with the MyotonPro (ICC = 0.82–0.96) [23].

In a study conducted by Gokalp et al., 20 participants with idiopathic scoliosis and 20 healthy subjects were evaluated. The mean Cobb angle was 20.30 ± 8.52°. As a result, muscle stiffness was observed in patients with greater scoliosis compared to healthy subjects on both sides. No static differences were observed in muscle tone, but the elasticity of the latissimus dorsi and middle trapezius on the concave side was reduced [19]. Another study evaluated the biomechanical properties of paravertebral muscles in patients with AIS [24]. MyotonPro and elastomyography were used in the study to evaluate the biomechanics of thoracic paravertebral muscles on the concave and convex sides. A total of 40 adolescent patients with idiopathic scoliosis with an average Cobb angle of 32.8° were monitored. Muscle tone, stiffness, and the deformation/relaxation ratio on the concave side were significantly higher than those on the convex side. No statistically significant differences in muscle elasticity were observed when comparing the concave and convex sides. The tone and stiffness of the concave paravertebral muscles were higher than those on the convex side [24].

A study conducted in Beijing on 23 patients with AIS (3 men and 20 women) aimed at detecting paravertebral muscle tone, stiffness, relaxation time, deformation/relaxation ratio, and elasticity on the concave and convex sides of the scoliosis curve at several points: the apex of the curve and the upper and lower limits. Muscle stiffness on the concave side was significantly higher than on the convex side at all three points. No significant difference in elasticity was found between the muscles on the two sides [25].

Through our study we want to point out that the possible differences observed in muscle stiffness between the concave and convex sides of scoliosis curves may serve as a potential guide for muscle stimulation and treatment efficacy. Although the main cause of biomechanical asymmetry of the paravertebral muscles between the two sides remains a challenge, the current study can help us to evaluate and recognize adolescent idiopathic scoliosis more comprehensively and to adapt the rehabilitation program according to the particularities of each patient.

Regarding the assessment of static plantar pressure and stabilometry, in the current study we aim to compare plantar pressure loadings on the feet in different testing conditions (eyes open, eyes closed, and head retroflexed). We predict an improvement in stabilometric parameters (length described by the center of pressure, area of the center of pressure ellipse, and speed of the center of pressure) after the 3-month exercise program. The study of Koura et al. highlighted the association between proprioceptive deficits and postural instability in individuals with scoliosis. They stated that the role of proprioceptive training in rehabilitation is a promising avenue for future research [26].

The PoData is a capacitive pressure distribution system. The study of van Gulick et al. pointed out that a wide range of static balance parameters can be reliably obtained in clinical settings when using different capacitive pressure distribution systems [27].

Concerning the limitations of available studies in this field to date, most of them involved small cohorts, which limits statistical power and the generalizability of the findings. Several of them lacked a control group, making it difficult to clearly isolate the effects of the exercise intervention. This limitation may be partially justified by ethical and practical concerns, as withholding or delaying an exercise regimen in a subset of AIS patients may not be feasible. Nevertheless, this issue could be addressed through well-designed prospective observational studies, in which patients are systematically assessed for adherence to the prescribed regimen, including both the frequency and consistency of compliance, allowing for indirect comparisons based on exposure levels.

The main limitations of this study include potential noncompliance with the physical exercise program, voluntary withdrawal of participants, and loss of subjects during the study, as well as the relatively small sample size. Operator-related errors and variations in the applied force during myotonometry also represent potential sources of bias; therefore, maintaining uniform conditions among participants and ensuring a relaxed environment during assessment are recommended. The lack of a control group (AIS patients with no physical exercise program) makes it difficult to attribute the observed changes exclusively to the exercise program. The improvements could have been influenced by external factors such as natural recovery, skeletal growth, or concomitant lifestyle changes. Despite these limitations reducing the credibility of the results and preventing the establishment of a definitive causal relationship between intervention and results, they cannot be easily overcome, as previously explained, due to impossibility of deferring treatment, as we do not consider it ethical to have a control group of AIS patients that will not receive the recommendation of physical exercise.

The strength of the current research lies in an association of assessment of myotonometric, plantar pressure, and stabilometric parameters pre- and post-intervention, offering a comprehensive view of biomechanical adaptations that has not to date been comprehensively described in the scientific literature.

## 5. Conclusions

We expect to record a difference between the muscles on the concave side and the convex side in terms of myotonometric parameters (resting muscle tone, elasticity, muscle stiffness, and muscle relaxation time). We also expect differences between the initial assessment and the assessment at 3 months (increased resting muscle tone, increased elasticity, and decreased stiffness at the 3-month assessment). The MyotonPro technique could be utilized to monitor changes in the biomechanics of the paraspinal muscle during the progression of curvature and evaluate the effectiveness of different therapies aimed at reducing muscle stiffness in adolescent idiopathic scoliosis. We predict an improvement in stabilometric parameters (length described by the center of pressure, area of the center of pressure ellipse, and speed of the center of pressure) after 3 months of physical exercise program.

## Data Availability

No new data were created or analyzed in this study.

## Supplementary Materials

The following supporting information can be downloaded at https://www.mdpi.com/article/10.3390/life16010101/s1, The TIDieR (Template for Intervention Description and Replication) Checklist.

## Abbreviations

The following abbreviations are used in this manuscript:

SRS	Scoliosis Research Society
AIS	Adolescent idiopathic scoliosis
